# Serum ferritin thresholds for the diagnosis of iron deficiency in pregnancy: a systematic review

**DOI:** 10.1111/tme.12408

**Published:** 2017-04-20

**Authors:** J. Daru, J. Allotey, J. P. Peña‐Rosas, K. S. Khan

**Affiliations:** ^1^ Women's Health Research Unit Centre for Primary Care and Public Health, Queen Mary University of London London UK; ^2^ Evidence and Programme Guidance, Department of Nutrition for Health and Development World Health Organization Geneva Switzerland

**Keywords:** iron deficiency, pregnancy, serum ferritin

## Abstract

The aim of this review was to understand the landscape of serum ferritin in diagnosing iron deficiency in the aetiology of anaemia in pregnancy. Iron deficiency in pregnancy is a major public health problem leading to the development of anaemia. Reducing the global prevalence of anaemia in women of reproductive age is a 2025 global nutrition target. Bone marrow aspiration is the gold standard test for iron deficiency but requires an invasive procedure; therefore, serum ferritin is the most clinically useful test. We undertook a systematic search of electronic databases and trial registers from inception to January 2016. Studies of iron or micronutrient supplementation in pregnancy with pre‐defined serum ferritin thresholds were included. Two independent reviewers selected studies, extracted data and assessed quality. There were 76 relevant studies mainly of observational study design (57%). The most commonly used thresholds of serum ferritin for the diagnosis of iron deficiency were <12 and <15 ng mL^−1^ (68%). Most primary studies provided no justification for the choice of serum ferritin threshold used, but 25 studies (33%) used thresholds defined by expert consensus in a guideline development process. There were five studies (7%) using a serum ferritin threshold defining iron deficiency derived from primary studies of bone marrow aspiration. Unified international thresholds of iron deficiency for women throughout pregnancy are required for accurate assessments of the global disease burden and for evaluating effectiveness of interventions addressing this problem.

**What is known about the topic?**

- There is variation in serum ferritin thresholds used to define iron deficiency in pregnancy, a condition with a large global burden of disease.

**What is new?**

- This variation leads to challenges in interpreting results of clinical interventions managing iron deficiency in pregnancy.

**What are the future key questions for work on the topic?**

- Accurate and internationally unified definitions of iron deficiency using serum ferritin in pregnancy are required.

Iron deficiency is the most common micronutrient deficiency in the world and the most common cause of anaemia (Kassebaum, 2016). A 50% reduction of anaemia in women of reproductive age by the year 2025 is a global nutrition target (World Health Organization, 2014). Iron deficiency anaemia (IDA) was also the most common cause of anaemia‐related disability in 2013 (Kassebaum, 2016). IDA affects one in four pregnancies in Europe (McLean *et al*., 2009; Scholl, 2010). Globally, the prevalence of anaemia in pregnant women in 2011 was estimated to be 38%, equivalent to 32 million women (Stevens *et al*., 2013). Prevalence of iron deficiency in the absence of anaemia is estimated to be between 30 and 60% (Daru *et al*., 2016). As erythropoiesis becomes more and more compromised, iron stores progress from being replete to reduced and finally depleted, resulting in anaemia (Scholl, 2005; Pavord *et al*., 2012). Both anaemic and non‐anaemic iron deficiencies in pregnancy have consequences for both the mother and their offspring (Scholl, 2011; Acosta *et al*., 2014). There are data demonstrating long‐term impairments in cognitive development and growth in babies exposed to IDA *in utero* (Congdon *et al*., 2012). Therefore, prevention and treatment of anaemia and iron deficiency is a priority to clinicians and policy makers in all countries (World Health Organization, 2011).

The traditional gold standard for defining iron deficiency is based on the absence of staining for haemosiderin on bone marrow aspirate viewed under microscopy, demonstrating a lack of iron available for erythropoiesis (Gale *et al*., 1963). This requires an invasive procedure not suitable for routine use. There are multiple biomarkers available for determining iron status by sampling peripheral blood. Of these, serum ferritin is the most clinically applicable in pregnancy (Pavord *et al*., 2012). There is variation in serum ferritin thresholds used to define iron deficiency in primary studies and in different policy documents (MMWR, 1998; World Health Organization, 2001). This makes it difficult to understand the scale of iron deficiency and interpret the effectiveness of interventions both at individual and population levels (Garcia‐Casal *et al*., 2014).

This systematic review was prospectively designed to investigate the range and characteristics of the thresholds of serum ferritin used to diagnose iron deficiency in pregnant women, used in primary intervention studies and national and international guidelines.

## MATERIALS AND METHODS

This review was designed to describe the current landscape of serum ferritin use in defining iron deficiency in pregnancy. It was prospectively registered (PROSPERO CRD42016037414) and was conducted and reported in compliance with the Preferred Reporting Items for Systematic Reviews (PRISMA) guidelines (Moher *et al*., 2009).

### Data sources and study selection

A database search, without language restrictions, was performed from conception to January 2016 on EMBASE, MEDLINE, The Cochrane Library, CINAHL, AMED and SIGLE. Searches of the following regional and international databases were also conducted in August 2015: CENTRAL, Web of Science, POPLINE, Open Grey, DARE, IBECS (http://ibecs.isciii.es/), Scielo (http://www.scielo.br/), WHO Global Index Medicus (GIM): African Index and Medicus (AIM), Index Medicus for the WHO Eastern, Mediterranean Region (IMEMR), LILACS, Pan American Health Library (PAHO), Western Pacific Region Index Medicus (WPRIM), Index Medicus for South‐East Asia Region (IMSEAR) and the WHO Library (WHOLIS). Searches aimed to identify all studies of iron or multiple micronutrient supplementation of any study design without language or date restrictions. The following subject headings and the key words, including ‘ferritin’, ‘ferritins’, ‘serum’, ‘plasma’, ‘diagnosis’ and ‘diagnostic’, were used (Appendix S1, Supporting Information).

Studies were selected using a two‐stage process. First, two independent reviewers (J. D. and J. A.) screened the titles and abstracts to identify eligible studies; this was followed by retrieval and assessment of the full‐text manuscripts of relevant citations. Any disagreements were resolved after discussion with a third reviewer (K. S. K.). We included studies with any design, conducted on pregnant or post‐partum women, carrying singleton or multiple pregnancies, where pre‐defined thresholds of serum ferritin for iron deficiency were used. We included studies with any laboratory ferritin assay used. Studies were excluded if authors had not pre‐specified values for iron deficiency according to serum ferritin concentrations prior to administering an intervention but had selected a value based on response to treatment. A *κ* coefficient of inter‐rater reliability was calculated (Landis and Koch, 1977).

### Data extraction and analysis

Data from selected studies were independently extracted by two reviewers (J. D. and J. A.) using a piloted data extraction form. Data were collected on the rationale for the threshold‐defining iron deficiency used, study design, geographical location of the studies and whether these study locations were in areas where soil‐transmitted helminthiasis or malaria infections were endemic. Because of the nature of this review, data are presented and analysed descriptively as meta‐analysis was not possible (Higgins, 2012). The results were tabulated, and all serum ferritin thresholds were converted to nanogram per millilitre to allow ease of comparison.

### Quality assessment

We used first principles to develop a strategy to objectively assess the quality of primary studies included in this review. The quality assessment tool contained six prompt questions. These were developed in collaboration with four experienced reviewers, international policy makers and clinicians from varied backgrounds. Two iterations of these prompt questions were required before the tool was finalised. The tool was piloted before being implemented in this review. The prompt questions and scoring system are outlined below. A score of 3 or more points was considered high quality. The maximum possible score achievable was 5, with a minimum score of 1 based on our inclusion and exclusion criteria.

1. Did the authors use a pre‐specified (*a priori*) threshold for serum ferritin in the study? (1 point).
2. Was a published reference for the pre‐specified threshold provided? (1 point).
3. Was the threshold derived from a comparison with the existing gold standard test (bone marrow aspirate) (1 point).
4. If not derived from a comparison with an existing gold standard test, was the threshold defined by a consensus of experts for any of the following (Jones and Hunter, 1995):
  - From a guideline development process (Rycroft‐Malone, 2001) (1 point)
  - Based on patient‐reported outcomes (Man‐Son‐Hing *et al*., 2000) (1/2 point)
  - Based on statistical analysis of existing data (Rycroft‐Malone, 2001) (1/2 point)
5. Does the selected threshold correlate to improved clinical outcomes (Grimshaw and Russell, 1993) (1 point).
6. Did the authors consider factors other than the disease of interest that may influence measurement of serum ferritin (e.g. multiple pregnancies, malaria, soil‐transmitted helminthiasis) (Bothwell, 2000; Mwangi *et al*., 2015)? (1 point).

## RESULTS

### Search results

**Figure 1:**
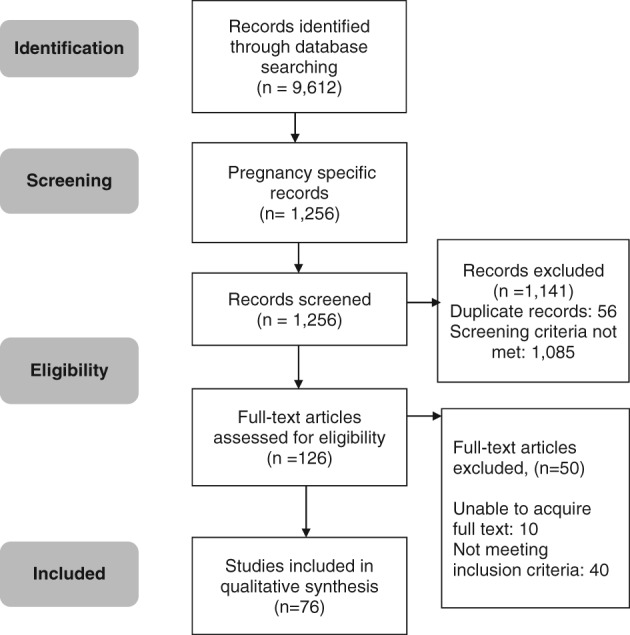
Flow chart of studies included in this systematic review of serum ferritin thresholds defining iron deficiency in pregnancy.

There were 9612 citations identified following the two electronic searches. Of these, 1256 citations were pregnancy‐specific. After screening titles and abstracts, 126 citations were eligible for full‐text review. Finally, 76 studies were included in the systematic review (Fig. 1). The *κ* statistic was 0·88, demonstrating excellent agreement between reviewers (Higgins, 2012).

### Study characteristics

Of the 76 included studies, 29 (38%) were conducted in women carrying singleton pregnancies (Table S1); the remaining were carried out in a mixed population of singleton and multiple pregnancies. A total of 24 (32%) were conducted in countries where parasitic infections, including malaria and/or hookworm infection, were endemic.

**Figure 2:**
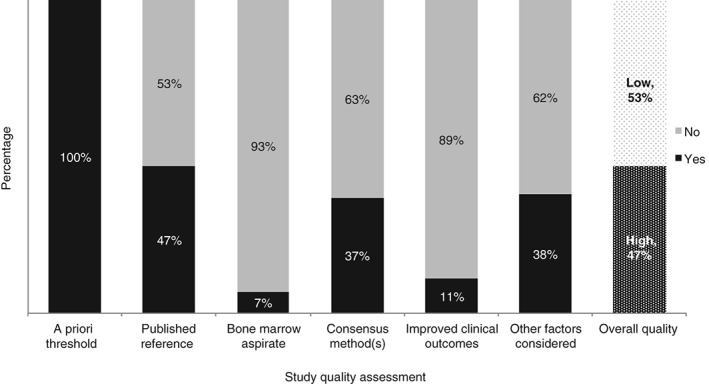
Quality assessment of studies included in the review of serum ferritin thresholds defining iron deficiency in pregnancy. Based on a quality assessment tool comprising of six questions described in the Methods section.

The studies included in the review were of varying methodological quality (Fig. 2). Of the included studies, 47% (36 studies) were of good quality, scoring 3 or more points on the quality assessment tool. All of the studies included in the review provided an a priori threshold of serum ferritin‐defining iron deficiency. Only 47% (36 studies) provided a reference to the source from which the threshold was derived (Fig. 2). There were five studies (7%) where the choice of serum ferritin threshold was based on a published primary study comparing serum ferritin measurements with bone marrow aspirates (Fig. 2). There were 28 studies (37%) where consensus methods were used to determine the threshold of serum ferritin used in the intervention study. Of these, 25 (33%) utilised consensus methods used in guideline development processes, one using consensus methods to develop patient‐reported outcomes and two using statistical analysis of existing data sets. There were six studies (8%) that used improvements in clinical outcomes (such as improvements in anthropometric measurements and nutritional status, neonatal birth weight and gestational age at delivery and iron endowment as measured by cord ferritin) following an intervention to identify the threshold of serum ferritin used to best define iron deficiency. There were 31 studies (41%) that considered the impact of external factors on the threshold used to define iron deficiency. This included factors such as multiple pregnancy and/or parasitic infections, including malaria (known to cause an acute phase reaction and rise in serum ferritin) or hookworm infestation (known to cause intestinal disease, increasing blood losses).

### Study methodology

**Figure 3:**
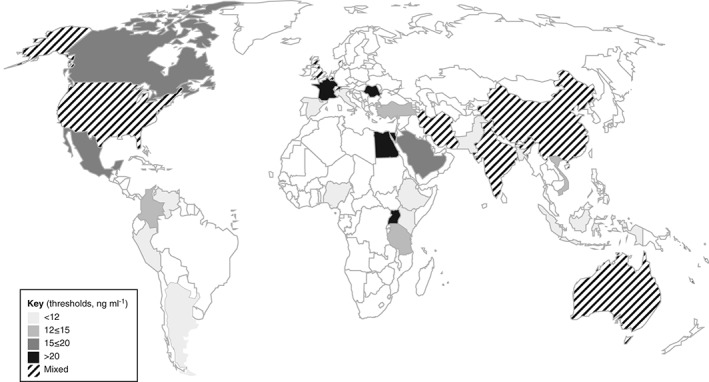
World map demonstrating the geographical distribution of serum ferritin thresholds used in studies of iron or multiple micronutrient supplementation in pregnancy. Shaded areas demonstrate countries contributing data to this systematic review.

**Figure 4:**
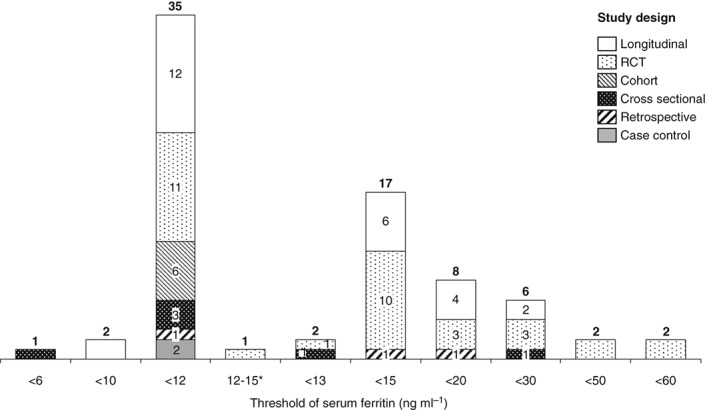
Histogram of serum ferritin (ng mL^−1^) thresholds defining iron deficiency in pregnancy, arranged by study design. *A threshold of <12 ng mL^−1^ for antenatal women and <15 ng mL^−1^ for post‐natal women was used in this study.

The largest number of individual studies of iron interventions were conducted in India (15 in total), followed by China (5 in total) (Fig. 3). There were several areas of sub‐Saharan Africa where no studies meeting the review criteria were found, similar to parts of Eastern Europe, Russia and South America. The most frequently used study design was longitudinal, including longitudinal cohort design (Fig. 4). The most commonly used thresholds of serum ferritin to diagnose iron deficiency in studies of iron and/or micronutrient supplementation in pregnant women were serum ferritin levels <12 (35 studies) and <15 ng mL^−1^ (17 studies). Other commonly used thresholds defining iron deficiency in pregnancy included serum ferritin <20 (8 studies) and <30 ng mL^−1^ (6 studies). Other serum ferritin thresholds (<6, <10, <50, and <60 ng mL^−1^) were used less commonly.

There were 40 studies that did not provide any justification or reference for the choice of serum ferritin threshold used (Fig. 2). Of the remaining studies that did, 10 studies cited the World Health Organization (WHO) 2001 guidelines as the reference for the threshold used; 9 studies referenced the Centres for Disease Control and Prevention (CDC) 1998 guidelines (MMWR, 1998) as the reference for the threshold used; 8 of the included studies referenced primary studies, including comparing serum ferritin levels with bone marrow aspirates taken from pregnant populations; and/or studies deriving thresholds from statistical analyses of laboratory reference ranges. The remaining studies included in this review referred to national guidelines and other published literature reviews as references for the thresholds defining iron deficiency used.

## DISCUSSION

### Main findings

This systematic review demonstrates the variation in the serum ferritin thresholds used to diagnose iron deficiency in pregnancy in research settings in the published literature. This variation was seen across a variety of study designs assessing the effects of iron interventions in different populations and geographical areas. Owing to the paucity of published data, a review of the diagnostic accuracy of serum ferritin thresholds defining iron deficiency in pregnancy is not possible. The data in this review demonstrate that a consensus on a particular threshold of serum ferritin for defining iron deficiency is lacking. The variation in thresholds leads to uncertainty regarding the diagnosis and optimal treatment of iron deficiency in pregnancy, with resultant consequences for both the mother and the offspring.

Iron deficiency and IDA increases the maternal risk of mortality secondary to haemorrhage (Cantwell *et al*., 2011) and sepsis (Bamber and Kinsella, 2015) with significant cost implications (Goonewardene *et al*., 2012). Severe maternal sepsis is the leading cause of maternal death in the UK, affecting 4·7 per 10 000 pregnancies (Acosta *et al*., 2014; Bamber and Kinsella, 2015). If anaemic women become septic, the risk of mortality is doubled (Acosta *et al*., 2014). Chronic fetal exposure to iron deficiency leads to low birth weight (Lone *et al*., 2004) and doubles the risk of pre‐term delivery (Beck *et al*., 2010). Moreover, there are emerging empirical data demonstrating that iron supplementation when iron stores are not depleted may also be associated with adverse maternal and fetal outcomes (Congdon *et al*., 2012; Siu, 2015), further highlighting the need for an accurate and unified threshold.

The adverse outcomes related to IDA are preventable by accurate diagnosis and timely treatment. The findings of this review show that the most frequently used thresholds for defining iron deficiency (<12 and <15 ng mL^−1^) are based on international guidelines informed by consensus meetings undertaken more than 15 years ago (MMWR, 1998; World Health Organization, 2001), not on published evidence.

### Strengths and limitations

This review describes the landscape of serum ferritin thresholds used in the diagnosis of iron deficiency in pregnant women, with a comprehensive summary of the existing thresholds in use. Although we attempted to identify thresholds used in unpublished and non‐peer‐reviewed sources by searching the grey literature and discussing the preliminary results with individuals with an interest in the subject, there is some risk of missing data. The data collected in this review were derived from a variety of study designs, and our investigation was centred on the rationale for the threshold investigators chose in studies of iron interventions in pregnancy. Investigating the use of other markers of iron status was beyond the scope of this review.

There are many markers of iron status (such as transferrin saturation [TSAT], soluble transferrin receptor [sTfR], hepcidin among others) that can be used to estimate iron status in the context of inflammation (Peyrin‐Biroulet *et al*., 2015) when serum ferritin levels are elevated but iron stores remain low. However, the use of these markers in diagnosing iron deficiency in pregnancy is not widespread (Pavord *et al*., 2012). This is because the impact of the physiological changes of pregnancy on measured levels of these blood markers have not been widely agreed upon (ACOG, 2008; Thomas *et al*., 2013).

The existing tools to assess the quality of studies (such as the Jadad and Newcastle‐Ottaway scales) were not appropriate in assessing studies included in this review. As a result, we developed a bespoke tool for assessing quality in primary studies of thresholds, defining a target condition in the absence of an existing gold standard. The strengths of our methods include using first principles to develop a quality assessment tool, (Crespi *et al*., 2014) a process used by other systematic reviewers to develop similarly novel quality assessment tools (Tirlapur *et al*., 2014). This tool was then reviewed and supported by external experts with the experience of interventional and diagnostic systematic reviews.

### Interpretation

Variation in international thresholds defining anaemia has been identified in the literature (New and Wirth, 2015). Discussions on a more targeted approach to unifying definitions not just for anaemia but iron deficiency and iron overload have ensued (Garcia‐Casal *et al*., 2014; Allard, 2015).

There are several physiological changes occurring in pregnancy that may contribute to the variation in thresholds of serum ferritin‐defining iron deficiency in pregnancy. These include (i) increased overall iron consumption compared to non‐pregnant states (Kaestel *et al*., 2015), (ii) second trimester plasma volume expansion (i.e. haemodilution); (Costantine, 2014), (iii) physiological rise in acute phase proteins secondary to pregnancy (Kaestel *et al*., 2015), (iv) changes in inflammatory measures in the final trimester of pregnancy (Viteri *et al*., 2012) and (v) the uncertainty in the degree of increased iron consumption and iron requirements in multiple pregnancies (Goodnight and Newman, 2009).

There are other external factors influencing iron consumption and therefore impacting the true threshold of serum ferritin representing iron deficiency in pregnancy. These include the effect of parasitic infections (such as malaria and hookworm infection) (Mwangi *et al*., 2015) and inflammatory conditions, including HIV (Clark *et al*., 2014), affecting the levels of serum ferritin (an acute phase protein) measured independent of body iron stores. The effect of these on measured serum ferritin levels in pregnant and non‐pregnant women is not clear, although steps are being taken to understand these complexities (Suchdev *et al*., 2016).

On reviewing the studies and national guidance referenced in the studies included in this review, a paper published in the British Journal of Haematology in 1998 was found to be the most frequently cited study for choice of serum ferritin threshold (van den Broek *et al*., 1998). In the study, van den Broek *et al*. (1998) compared bone marrow aspirates samples of 93 Malawian pregnant women with serum ferritin levels from peripheral blood samples and found that a serum ferritin threshold of <30 ng mL^−1^ had a sensitivity of 90% and a specificity of 85·1% in identifying individuals with absent iron stores on bone marrow staining in pregnancy (van den Broek *et al*., 1998). In this study, 44 of the 93 patients were HIV positive. The authors attempted to correct for the impact of HIV infection by adjusting the results based on C‐reactive protein (CRP) levels. HIV infection is an independent contributor to iron deficiency (Minchella *et al*., 2015). The effect of HIV status of included women in the study by van den Broek *et al*. (1998) on the serum ferritin estimates cannot be accurately estimated because of the small sample size (private communication). Nevertheless, this study continues to be used as a bench mark for defining iron deficiency and is used as a method of justifying selecting a serum ferritin threshold of <30 ng mL^−1^ defining iron deficiency in numerous studies of iron interventions and national guidelines (Sukrat and Sirichotiyakul, 2006; Ndyomugyenyi *et al*., 2008; Shields *et al*., 2011; Pavord *et al*., 2012). After consulting national guideline authors, senior obstetricians and haematologists in the UK, we found that a threshold‐defining iron deficiency of <30 ng mL^−1^ in pregnancy is widely used in practice to guide therapy.

There are multiple measures that can be taken to establish a unified, defining threshold for a target condition in the absence of an established gold standard test threshold. A Health Technology Assessment (HTA) report published in 2007 outlines the process of the test validation paradigm (Rutjes *et al*., 2007). This involves using consensus methods to establish a threshold from clinicians, scientists and policy makers followed by continual assessment in new primary studies or existing data sets on whether the threshold improves clinical outcomes for patients and/or is cost effective (Rutjes *et al*., 2007).

Choosing a threshold based on consensus methods may be challenging as a consensus between international experts on this matter does not currently exist. The arguments for and against using a more conservative threshold for defining iron deficiency in pregnancy are multi‐fold. A higher threshold (such as <30 ng mL^−1^) may allow for issues surrounding the uncertainty of iron consumption in pregnancy (singleton and multiple), pregnancy‐related pro‐inflammatory changes and the effect of physiological plasma volume expansion to be considered (Costantine, 2014). However, a higher threshold can increase the burden of the disease in countries where a very high prevalence already exists (Garcia‐Casal *et al*., 2014), also increasing the economic burden for policy makers.

Good‐quality primary studies exploring the optimal serum ferritin threshold defining iron deficiency in pregnancy are required. The possibility of deriving a threshold using pre‐existing data from primary studies is an option (Suchdev *et al*., 2016). Databases and individual patient data meta‐analyses may be helpful in providing extra information to determine the optimum threshold defining iron deficiency. However, care must be taken to ensure that thresholds derived from novel primary studies or statistical analyses are representative of the target condition and correlate to clinical outcomes.

## CONCLUSION

The current published literature, including national guidelines and primary studies of iron interventions, showed variation in defining the ferritin threshold of iron deficiency in pregnancy. A unified definition of iron deficiency is required.

## CONFLICT OF INTEREST

The authors alone are responsible for the views expressed in this article, and they do not necessarily represent the views, decisions or policies of the institutions with which they are affiliated.

The authors declare no conflicts of interest.

## Supporting information


**Appendix S1.** Searches undertaken until January 2016 on EMBASE, MEDLINE, The Cochrane Library, CINAHL, AMED and SIGLE using the following mesh headings.

**Table S1.** Table of characteristics of studies of iron or multiple micronutrient supplementation included in the systematic review of serum ferritin thresholds used in pregnancy. RCT, randomised controlled trial; CDC, Centres for Disease Control; WHO, World Health Organization; Y, yes, N, no.
