# Supplementary material for: Serum ferritin thresholds for the diagnosis of iron deficiency in pregnancy: a systematic review
Source: Transfus Med. 2017 Apr 20;27(3):167–74. doi: 10.1111/tme.12408 (PMC5763396; doi:10.1111/tme.12408)
Supplement: Supplementary file 1 — Appendix S1. Searches undertaken until January 2016 on EMBASE, MEDLINE, The Cochrane Library, CINAHL, AMED and SIGLE using the following mesh headings. [file TME-27-167-s002.docx]

**Appendix S1**

**Searches undertaken until January 2016 on EMBASE, MEDLINE, The Cochrane Library, CINAHL, AMED and SIGLE using the following mesh headings.**

#1. pregnancy [mesh] pregn* Pregnant woman [mesh]

#2. iron deficiency [mesh] iron defic*

#3. Hemoglobin [mesh] hemoglob*

#4. Blood cell count [mesh] full blood count [mesh]

#5. Ferritin [mesh] serum ferrit*

#6. Anemia [mesh] anaem* Anemia [mesh] anem*

**Searches undertaken until August 2015 by the WHO secretariat:**

**MEDLINE**

1. exp Iron Metabolism Disorders/

2. Iron, Dietary/

3. ((iron or ferrous* or ferric* or fe) adj5 (deficien* or overload* or excess*)).tw.

4. or/1-3

5. exp Ferritins/

6. ferritin*.tw.

7. isoferritin*.tw.

8. or/5-7

9. exp Biological Markers/

10. marker*.tw.

11. biomarker*.tw.

12. screen*.tw.

13. detect*.tw.

14. accura*.tw.

15. predict*.tw.

16. diagnos$.ti,ab.

17. Immunoassay/

18. (immunoassay* or assay*).tw.

19. immunoturbidimetric.tw.

20. immuno turbidimetric.tw.

21. Latex Fixation Tests/

22. latex agglutination.tw.

23. (SPECIFICITY or SPECIFICITIES).tw.

24. (SENSITIVITY or SENSITIVE or SENSITIVITIES).tw.

25. "Sensitivity and Specificity"/ and (Plasma/ or plasma*.tw.)

26. ROC Curve/ and (Plasma/ or plasma*.tw.)

27. ROC.ab. and (Plasma/ or plasma*.tw.)

28. Area Under Curve/

29. AUC.ab.

30. or/9-29

31. 4 and 8 and (30 or exp Ferritin/bl)

32. limit 31 to humans

**EMBASE (OVID)**

1. exp iron metabolism disorder/

2. iron intake/

3. ((iron or ferrous* or ferric* or fe) adj5 (deficien* or overload* or excess*)).tw.

4. or/1-3

5. exp ferritin/

6. ferritin*.tw.

7. isoferritin*.tw.

8. or/5-7

9. exp biological marker/

10. marker*.tw.

11. biomarker*.tw.

12. screen*.tw.

13. detect*.tw.

14. accura*.tw.

15. predict*.tw.

16. diagnos*.ti,ab.

17. immunoassay/

18. (immunoassay* or assay*).tw.

19. immunoturbidimetric.tw.

20. immuno turbidimetric.tw.

21. latex agglutination test/

22. latex agglutination.tw.

23. (SPECIFICITY or SPECIFICITIES).tw.

24. (SENSITIVITY or SENSITIVE or SENSITIVITIES).tw.

25. "Sensitivity and Specificity"/ and (Plasma/ or plasma*.tw.)

26. ROC Curve/ and (Plasma/ or plasma*.tw.)

27. ROC.ab. and (Plasma/ or plasma*.tw.)

28. Area Under Curve/

29. AUC.ab.

30. or/9-29

31. 4 and 8 and 30

32. limit 31 to (human and embase)

**CINAHL (EBSCO)**

S35 S3 AND S7 AND S34

S34 (S8 OR S9 OR S10 OR S11 OR S12 OR S13 OR S14 OR S15 OR S16 OR S17 OR S18 OR S19 OR S20 OR S21 OR S22 OR S27 OR S29 OR S31 OR S32 OR S33)

S33 AB area under curve

S32 AB AUC

S31 S26 AND S30

S30 AB ROC

S29 S26 AND S28

S28 (MH "ROC Curve")

S27 S23 AND S26

S26 S24 OR S25

S25 plasma

S24 (MH "Plasma")

S23 (MH "Sensitivity and Specificity")

S22 (SENSITIVITY or SENSITIVE or SENSITIVITIES)

S21 (SPECIFICITY or SPECIFICITIES)

S20 latex agglutination

S19 immuno turbidimetric

S18 immunoturbidimetric

S17 (immunoassay* or assay*)

S16 (MH "Immunoassay")

S15 AB diagnos* OR TI diagnos*

S14 predict*

S13 accura*

S12 detect*

S11 screen*

S10 biomarker*

S9 marker*

S8 (MH "Biological Markers+")

S7 S4 OR S5 OR S6

S6 isoferritin*

S5 ferritin*

S4 (MH "Ferritin")

S3 S1 OR S2

S2 ((iron or ferrous* or ferric* or fe) N5 (deficien* or overload* or excess*))

S1 (MH "Iron Metabolism Disorders+")

**Web of Science (SCI, SSCI, CPCI & CRCI-SSH)**

#16 #15 AND #2 AND #1

DocType=All document types; Language=All languages;

#15 #14 OR #13 OR #12 OR #11 OR #10 OR #9 OR #8 OR #7 OR #6 OR #5 OR #4 OR #3

DocType=All document types; Language=All languages;

#14 TS=(AUC or "area under curve")

DocType=All document types; Language=All languages;

#13 #12 AND #11

DocType=All document types; Language=All languages;

#12 TS=(ROC)

DocType=All document types; Language=All languages;

#11 TOPIC: (plasma*)

DocType=All document types; Language=All languages;

#10 TOPIC: ((SENSITIVITY or SENSITIVE or SENSITIVITIES))

DocType=All document types; Language=All languages;

#9 TOPIC: ((SPECIFICITY or SPECIFICITIES))

DocType=All document types; Language=All languages;

#8 TOPIC: (latex agglutination)

DocType=All document types; Language=All languages;

#7 TOPIC: (immunoturbidimetric or "immuno turbidimetric")

DocType=All document types; Language=All languages;

#6 TOPIC: ((immunoassay* or assay*))

DocType=All document types; Language=All languages;

#5 TOPIC: (detect* or accura* or predict* or diagnos*)

DocType=All document types; Language=All languages;

#4 TOPIC: (screen*)

DocType=All document types; Language=All languages;

#3 TOPIC: (marker* or biomarker*)

DocType=All document types; Language=All languages;

#2 TOPIC: (ferritin* or isoferritin*)

DocType=All document types; Language=All languages;

#1 TOPIC: (((iron or ferrous* or ferric* or fe) near/5 (deficien* or overload* or excess*)))

DocType=All document types; Language=All languages;

**POPLINE and Open Grey**

(ferritin OR isoferritin) AND (iron* OR ferrous* OR ferric* OR fe) AND (deficien* OR overload* OR excess*) AND (marker* OR biomarker* OR screen* OR detect* OR accura* OR predict* OR diagnos* OR immunoassay* OR assay* OR immunoturbidimetric OR "immuno turbidimetric" OR "latex agglutination" OR SPECIFICITY OR (SPECIFICITIES OR SENSITIVITY OR SENSITIVE OR SENSITIVITIES AND plasma*))

**TRoPHI and Bibliomap**

1. Freetext: "marker*" OR "biomarker*" OR "screen*" OR "detect*" OR "accura*" OR "predict*" OR "diagnos*" OR "immunoassay*" OR "assay*" OR "immunoturbidimetric" OR "immuno turbidimetric" OR "latex agglutination" OR ("SPECIFICITY" OR "SPECIFICITIES" OR "SENSITIVITY "OR "SENSITIVE" OR "SENSITIVITIES" AND "plasma*")

2. Freetext: "ferritin" OR "isoferritin"

3. Freetext: "iron*" OR "ferrous*" OR "ferric*" OR "fe"

4. 1 AND 2 AND 3

**IBECS, PAHO, WHOLIS,** **EMRO, AFRO and LILACS (BIRME)**

ferritin$ or isoferritin$ [Words] and (iron or ferrous$ or ferric$ or fe) and (deficien$ or overload$ or excess$) [Words] and marker$ or biomarker$ or screen$ or detect$ or accura$ or predict$ or diagnos$ or immunoassay$ or assay$ or immunoturbidimetric or immuno turbidimetric or latex agglutination or SPECIFICITY or SPECIFICITIES or SENSITIVITY or SENSITIVE or SENSITIVITIES or ROC or AOC [Words]

**SCIELO**

(ferritin$ or isoferritin$) AND (iron or ferrous$ or ferric$ or fe) AND ((deficien$ or overload$ or excess$) and (marker$ or biomarker$ or screen$ or detect$ or accura$ or predict$ or diagnos$ or immunoassay$ or assay$ or immunoturbidimetric or immuno turbidimetric or latex agglutination or SPECIFICITY or SPECIFICITIES or SENSITIVITY or SENSITIVE or SENSITIVITIES or ROC or AOC))

**WPRO, IMSEAR (GLOBAL INDEX MEDICUS)**

(ferritin* or isoferritin*) AND (iron or ferrous* or ferric* or fe) AND ((deficien* or overload* or excess*) and (marker* or biomarker* or screen* or detect* or accura* or predict* or diagnos* or immunoassay* or assay* or immunoturbidimetric or immuno turbidimetric or latex agglutination or SPECIFICITY or SPECIFICITIES or SENSITIVITY or SENSITIVE or SENSITIVITIES or ROC or AOC))

**INMED**

ferritin or isoferritin AND iron or ferrous or ferric or fe AND marker or biomarker or screen or detect or accuracy or predict or diagnose or immunoassay or assay or immunoturbidimetric or immuno turbidimetric or latex agglutination or SPECIFICITY or SPECIFICITIES or SENSITIVITY or SENSITIVE or SENSITIVITIES or ROC or AOC

**Native Health Research database**

ferritin
