# Supplementary material for: Serum ferritin thresholds for the diagnosis of iron deficiency in pregnancy: a systematic review
Source: Transfus Med. 2017 Apr 20;27(3):167–74. doi: 10.1111/tme.12408 (PMC5763396; doi:10.1111/tme.12408)
Supplement: Supplementary file 2 — Table S1. Table of characteristics of studies of iron or multiple micronutrient supplementation included in the systematic review of serum ferritin thresholds used in pregnancy. RCT, randomised controlled trial; CDC, Centres for Disease Control; WHO, World Health Organization; Y, yes, N, no. [file TME-27-167-s001.docx]

| **Study Authors** | **Study design** | **Threshold used** | **Rationale for threshold** | **Country of origin area of endemic parasitic infection (Y/N)** | **Source of threshold** |
| --- | --- | --- | --- | --- | --- |
| **WOMEN WITH SINGLETON AND MULTIPLE PREGNANCIES** | | |  |  |  |
| Aggawal, 2012 | RCT | <15ng ml^-1^ | No justification | India, Y | None found |
| Akpanabiatu, 1998 | Longitudinal | <10ng ml^-1^ | Primary study of bone marrow aspirate | Nigeria, Y | None found |
| Al-Moumen, 1996 | Longitudinal | <20ng ml^-1^ | No justification | Saudi Arabia, N | None found |
| Alper, 2000 | Longitudinal | <12ng ml^-1^ | No justification | Lebanon, N | None found |
| Alusala, 2008 | Longitudinal | <12ng ml^-1^ | No justification | Kenya, Y | None found |
| Arija, 2013 | Longitudinal | <12ng ml^-1^ | CDC criteria | Spain, N | CDC 1998 consensus |
| Arturo, 2002 | Longitudinal | <12ng ml^-1^ | No justification | Venezuela, N | None found |
| Ayub, 2008 | Longitudinal | <12ng ml^-1^ | No justification | Pakistan, Y | None found |
| Baraka, 2011 | Cross sectional | <13ng ml^-1^ | Primary study of serum ferritin thresholds | Belgium, N | RCT of varying serum ferritin thresholds defining ID |
| Bencaiova, 2012 | Longitudinal | <20ng ml^-1^ | CDC criteria | Switzerland, N | CDC 1998 consensus |
| Breymann, 2001 | Longitudinal | <15ng ml^-1^ | No justification | Switzerland, N | None found |
| Casanova, 2005 | Longitudinal | <10ng ml^-1^ | Primary studies | USA, N | Three primary studies of iron supplementation |
| Casanueva, 2003 | Longitudinal | <20ng ml^-1^ | CDC criteria | Mexico, N | CDC 1998, consensus |
| Cogswell, 2003 | RCT | <20ng ml^-1^ | CDC criteria | USA, N | CDC 1998, consensus |
| Colomer, 1990 | Longitudinal | <12ng ml^-1^ | National guidelines | Spain, N | None found |
| Finklestein, 2014 | Longitudinal | <12ng ml^-1^ | No justification | India, Y | None found |
| Gibson, 2008 | Cross sectional | <12ng ml^-1^ | Primary study | Ethiopia, Y | Primary observational study of the local population |
| Halksworth, 2003 | Longitudinal | <15ng ml^-1^ | No justification | UK, N | None found |
| Hinderaker, 2002 | Longitudinal | <15ng ml^-1^ | Literature review | Tanzania, Y | Expert review of published evidence from 1989 |
| Hyder, 2004 | Longitudinal | <12ng ml^-1^ | National guidelines | Bangladesh, Y | National consensus meeting in 1990 |
| Kamdi, 2014 | RCT | <12ng ml^-1^ | National guidelines | India, Y | National consensus meeting in 2001 |
| Kaufer, 1990 | Longitudinal | <20ng ml^-1^ | No justification | Mexico, N | None found |
| Kosus, 2012 | Retrospective Case control | <15ng ml^-1^ | No justification | Turkey, N | None found |
| Kraft, 2005 | RCT | <15ng ml^-1^ | No justification | Switzerland, N | None found |
| Lindstrom, 2010 | Longitudinal | <12ng ml^-1^ | WHO Guidelines | Bangladesh, Y | WHO, 2001 consensus |
| Ma, 2010 | Cross sectional | <12ng ml^-1^ | Literature review | China, N | Published review |
| Malik, 2011 | RCT | <60ng ml^-1^ | Clinical haematology textbook | India, Y | Published expert opinion |
| Marti Carvel, 2002 | Cross sectional | <12ng ml^-1^ | WHO Guidelines | Venezuela, Y | WHO 2001 consensus |
| Massot, 2003 | Retrospective Case control | <12ng ml^-1^ | CDC criteria | Belgium, N | CDC 1998 consensus |
| McKenna, 2003 | RCT | <12ng ml^-1^ | No justification | UK, N | None found |
| Milman, 2005 | RCT | <12ng ml^-1^ (antenatal)  <15ng ml^-1^  (postnatal) | Primary study | Denmark, N | Prior studies of iron supplementation and dose response curves |
| Milman, 1989 | Longitudinal | <15ng ml^-1^ | No justification | Denmark, N | None found |
| Milman, 2014 | RCT | <15ng ml^-1^ | No justification | Denmark, N | None found |
| Ndyomugyeny, 2008 | Longitudinal | <30ng ml^-1^ | Primary study of bone marrow aspirates | Uganda, Y | Van den Broek et al. |
| Neeru, 2012 | RCT | <27ng ml^-1^ | Laboratory reference range | India, Y | Local laboratory reference range |
| Nurdiati, 2001 | Longitudinal Cohort | <12ng ml^-1^ | No justification | Indonesia, N | None found |
| Ortiz, 2011 | RCT | <15ng ml^-1^ | No justification | Argentina, N | None found |
| O’Brien, 2000 | Retrospective Case control | <20ng ml^-1^ | No justification | Canada, N | None found |
| Saha, 2007 | RCT | <12ng ml^-1^ | No justification | India, Y | None found |
| Sarkate, 2007 | RCT | <12ng ml^-1^ | No justification | India, Y | None found |
| Shields, 2011 | Cross sectional | <30ng ml^-1^ | National guidelines | UK, N | Van den Broek et al. |
| Singh, 1998 | Cross sectional | <6ng ml^-1^ | No justification | Singapore, N | None found |
| Singh, 1998 | RCT | <20ng ml^-1^ | No justification | Singapore, N | None found |
| Singh, 2013 | RCT | <60ng ml^-1^ | No justification | India, Y | None found |
| Tiawari, 2012 | Longitudinal | <12ng ml^-1^ | CDC criteria | India, Y | CDC 1998 consensus |
| Tran, 2013 | Longitudinal | <15ng ml^-1^ | WHO guidelines | Vietnam, N | WHO 2001 consensus |
| Thomsen, 1993 | RCT | <15ng ml^-1^ | No justification | Denmark, N | None found |
| Walsh, 2011 | Longitudinal | <12ng ml^-1^ | WHO guidelines | UK, N | WHO 2001 consensus |
| Zimmerman, 2007 | RCT | <15ng ml^-1^ | WHO guidelines | Switzerland | WHO 2001 consensus |
| **WOMEN WITH SINGLETON PREGNANCIES ONLY** | | | | | |
| Arija, 2013 | Longitudinal | <12ng ml^-1^ | CDC criteria | Spain, N | CDC 1998 consensus |
| Bayoumenu, 2002 | RCT | <50ng ml^-1^ | National guidance | France, N | Consensus from 1997 |
| Carriagia, 1991 | Longitudinal | <12ng ml^-1^ | No justification | USA, N | None found |
| Deeba, 2012 | RCT | <15ng ml^-1^ | No justification | India, Y | None found |
| Frossler, 2013 | RCT | <12ng ml^-1^ | WHO Guidelines | Australia, N | WHO 2001 consensus |
| Falahi, 2011 | RCT | <12ng ml^-1^ | No justification | Iran, N | None found |
| Gupta, 2014 | RCT | <15ng ml^-1^ | No justification | India, Y | None found |
| Khallafallah, 2010 | RCT | <30ng ml^-1^ | No justification | Tasmania, N | None found |
| Kochar, 2013 | RCT | <15ng ml^-1^ | No justification | India, Y | None found |
| Mehedintu, 2015 | RCT | <37ng ml^-1^ | No justification | Romania, N | None found |
| Mei, 2014 | RCT | <12ng ml^-1^ | CDC criteria | China, N | CDC, 1998 consensus |
| Mukhopadhyay, 2004 | RCT | <12ng ml^-1^ | No justification | India, Y | None found |
| Nair, 2014 | Longitudinal | <12ng ml^-1^ | No justification | India, Y | None found |
| Nappi, 2009 | RCT | <12ng ml^-1^ | No justification | Italy, N | None found |
| Perego, 2005 | Longitudinal Cohort | <12ng ml^-1^ | Primary study | Argentina, N | Local population based observational study |
| Rezk, 2016 | RCT | <25ng ml^-1^ | No justification | Egypt, Y | None found |
| Ribot, 2012 | Retrospective Case control | <12ng ml^-1^ | WHO Guidelines | Spain, N | WHO, 2001 consensus |
| Ribot, 2013 | Longitudinal | <12ng ml^-1^ | CDC criteria | Spain, N | CDC 1998, consensus |
| Ronnenberg, 2004 | Longitudinal | <12ng ml^-1^ | WHO guidelines | China, N | WHO, 2001 consensus |
| Rusia, 1999 | Longitudinal | <12ng ml^-1^ | No justification | India, Y | None found |
| Shao, 2012 | Longitudinal | <15ng ml^-1^ | Review | China, N | Review of literature from 1989 |
| Siega-Riz, 2006 | RCT | <20ng ml^-1^ | US Task Force literature review | USA, N | Review of literature from 1993 |
| Sukrat, 2006 | Longitudinal | <30ng ml^-1^ | Primary study of bone marrow aspirates | China, N | Van den Broek et al |
| Tariq, 2015 | RCT | <12ng ml^-1^ | No justification | Pakistan, Y | N None found |
| Verstraelen, 2005 | Retrospective Case control | <12ng ml^-1^ | WHO guidelines | Belgium, N | WWHO 2001, consensus |
| Zavletta, 2000 | RCT | <12ng ml^-1^ | WHO guidelines | Peru, N | WHO 2001, consensus |
| Ziaei, 2008 | RCT | <15ng ml^-1^ | CDC criteria | Iran, N | CDC 1998, consensus |

Table S1: Table of characteristics of studies of iron or multiple micronutrient supplementation included in systematic review of serum ferritin thresholds used in pregnancy. RCT- randomised controlled trial, CDC- Centres for Disease Control, WHO- World Health Organization, Y- yes, N- no
